# Genome-Wide Identification of *GRAS* Gene Family in *Cunninghamia lanceolata* and Expression Pattern Analysis of ClDELLA Protein Under Abiotic Stresses

**DOI:** 10.3390/ijms252212262

**Published:** 2024-11-15

**Authors:** Yi Luo, Mengshuang Jin, Junjie Yang, Ye Yang, Runxin Guo, Huan Luo, Tianhao Guo, Jin Xu

**Affiliations:** State Key Laboratory of Tree Genetics and Breeding, Co-Innovation Center for Sustainable Forestry in Southern China, College of Forestry and Grassland, Nanjing Forestry University, Nanjing 210037, China; luoyi@njfu.edu.cn (Y.L.); jms3213240278@163.com (M.J.); yangjj@njfu.edu.cn (J.Y.); y2yshikouyang@njfu.edu.cn (Y.Y.); 13509756791@163.com (R.G.); huanluo27@gmail.com (H.L.); 17855687576@163.com (T.G.)

**Keywords:** *Cunninghamia lanceolata*, GRAS gene family, DELLA, abiotic stresses

## Abstract

The Chinese fir (*Cunninghamia lanceolata)* is a significant species utilized in afforestation efforts in southern China. It is distinguished by its rapid growth and adaptability to diverse environmental conditions. The GRAS gene family comprises a group of plant-specific transcription factors that play a pivotal role in plant growth and development, response to adversity, and hormone regulatory networks. However, the exploration of the GRAS family in gymnosperm Chinese fir has not yet begun. In this study, a total of 43 *GRAS* genes were identified in the whole genome of Chinese fir, and a phylogenetic analysis classified them into nine distinct subfamilies. Gene structure analysis revealed that the majority of *ClGRAS* genes lacked introns. It is notable that among these proteins, both *ClGAI* and *ClGRA* possess distinctive DELLA structural domains. Cis-acting element analysis revealed that nearly all *ClGRAS* genes contained light-responsive elements, while hormone-responsive elements, environmental-responsive elements (low-temperature- or defense-responsive elements), and meristem-organization-related elements were also identified. Based on transcriptome data and RT-qPCR expression patterns, we analyzed the expression of *ClGAI* and *ClRGA* genes across different developmental stages, hormones, and three abiotic stresses. Subcellular localization analysis demonstrated that *ClGAI* and *ClRGA* were localized to the nucleus. Transcriptional activation assays showed that both genes have self-activating activity. In conclusion, the results of this study indicate that the *ClGRAS* gene family is involved in the response of Chinese fir to environmental stress. Further research on the *ClDELLA* genes provides valuable information for exploring the potential regulatory network of DELLA proteins in Chinese fir.

## 1. Introduction

The Chinese fir is a fast-growing coniferous species renowned for its exceptional wood properties and high wood productivity in southern China [1]. It is the most extensively cultivated tree species in southern China, with a history spanning over a thousand years. However, adverse environmental conditions, such as elevated temperatures and drought, can affect the growth and development of Chinese fir [2]. The GRAS transcription factors play a role in plant development and response to a variety of stresses, but the GRAS gene family has been rarely studied in Chinese fir.

Plant transcription factors (TFs) play a significant role in a multitude of biological processes [3]. TFs are not only integral components of plant signaling pathways but are also capable of participating in plant responses to biotic and abiotic stresses [4]. The GRAS family is named after three transcription factors: gibberellin insensitive factor (GAI), repressor of GA (RGA), and scarecrow (SCR) [5]. These TFs are typically distinguished by a highly conserved C-terminal structural domain, which belongs to the GRAS family of structural domains, and a variable N-terminal structural domain [6]. Based on the N-terminal length and sequence variation, GRAS is typically classified into 8–17 subfamilies, including LS, DELLA, SCL9, SCR, PAT1, SHR, HAM, SCL3, and others [7].

The advent of next-generation sequencing technologies has resulted in the generation of a substantial amount of data pertaining to genomic, transcriptomic, proteomic, and metabolomic processes, which has in turn led to the development of new analytical techniques capable of processing and interpreting these data. Consequently, GRAS gene families have been identified and analyzed in numerous plant species, including *Arabidopsis thaliana* [8], *Nicotiana tabacum* [9], *Populus tomentosa* [10], *Oryza sativa* [11], *Triticum aestivum* [12], *Actinidia chinensis* [13], *Eucalyptus megacephalus* [14], and *Manihot esculenta* [15]. As a consequence of the divergence in the N-terminal structural domains, the functions exhibit variation among subfamilies. It has been demonstrated that the PAT1 subfamily plays a multifaceted role in light signaling and transcriptional regulation [16]. In *A. thaliana*, three genes within the PAT1 subfamily, namely *PAT1*, *SCL5*, and *SCL21*, have been identified as positive regulators of light signaling [17]. The grape (*Vitis vinifera*) VaPAT1 transcription factor has been demonstrated to regulate jasmonic acid (JA) biosynthesis in response to grape cold stress [18]. *GhSCL13-2A*, a member of the *Gossypium hirsutum* PAT1 family, has been demonstrated to positively regulate resistance to *Botrytis cinerea* in cotton [19]. This is accomplished by modulating JA and salicylic acid (SA) signaling pathways, in addition to the accumulation of reactive oxygen species. The SCR and SHR form the SCR/SHR complex, which plays an important role in the radial organization of roots and stems [20]. It is worthy of note that the SCL3 subfamily and the DELLA subfamily exert opposing effects on GA signaling in plants [21]. Following gibberellin A3 (GA3) treatment, nine of the *LkGRAS* genes in *Larix kaempferi* exhibited upregulation [16].

The DELLA subfamily occupies a distinctive position within the GRAS family. All TFs in the subfamily possess a distinctive DELLA structural domain at their N-terminus [22]. Interestingly, the DELLA family derives its name from a short-chain amino acid, D-E-L-L-A [23], which is present in this conserved structural domain. It is noteworthy that DELLA proteins lack the typical DNA-binding structural domains observed in other proteins. Nevertheless, they have been demonstrated to interact with a variety of transcription factors, thereby influencing plant growth and development, as well as plant responses to diverse environmental stresses [24]. It is well established that DELLA proteins act as negative regulators of the gibberellin signaling pathway [25]. The gibberellin hormone plays a pivotal role in this process, whereby it triggers the polyubiquitination and subsequent degradation of DELLA proteins via the 26S proteasome. DELLA and JAZ proteins act synergistically to repress the transcriptional functions of *MYB21* and *MYB24*, thereby inhibiting fiber elongation [26]. DELLA proteins interact with FLC to inhibit perforation [27]. Moreover, DELLA proteins have been demonstrated to activate the JA defense pathway, and they bind to the MYC2 transcription factor [28]. In poplar, however, DELLA has been demonstrated to inhibit the promotion of vascular cambium by *ARK2* and *WOX4* [29]. In this study, we employed all the genomic data from the Chinese fir species to identify and analyze *GRAS* genes. Our analysis yielded a total of 43 *GRAS* genes. The phylogenetic relationships, chromosomal locations, gene structures, structural domains and conserved motifs, and cis-acting elements of the *ClDELLA* genes were also analyzed. Additionally, the expression patterns of *ClDELLAs* were investigated in different tissues, hormones, and abiotic stresses using transcriptome data and qRT-PCR. The findings presented here will prove invaluable for future research into the multifaceted roles of Chinese fir DELLA proteins.

## 2. Results

### 2.1. Identification and Physicochemical Characterization of Chinese Fir GRAS Gene Family

We screened and analyzed the whole genome of Chinese fir by BLAST comparison and HMM search. In total, 43 *ClGRAS* genes were identified by manual screening and named *ClGRAS1*~*ClGRAS41*, among which two *DELLA* genes were named *ClGAI* and *ClRGA* individually. In addition, the physicochemical properties of ClGRAS proteins were analyzed using the online tool Expasy (https://web.expasy.org/protparam/, accessed on 8 May 2024) (Table 1). According to the results in the table, the number of amino acids in ClGRAS proteins varied widely among them, with lengths ranging from 390 aa (*ClGRAS29*) to 1316 aa (*ClGRAS1*), with an average of 605 aa. The molecular weights (MW) of 43 genes ranged from 43.72 kDa (*ClGRAS29*) to 146.94 kDa (*ClGRAS1*), with an average of 67.55 kDa. The isoelectric point (pI) ranged from 4.46 (*ClGRAS35*) to 8.88 (*ClGRAS25*), with a total of 41 ClGRAS genes exhibiting pI values less than 7.0, indicative of acidic properties. Conversely, two genes, *ClGRAS24* and *ClGRAS25*, demonstrated pI values greater than 7.0, suggesting alkali characteristics. The aliphatic index of the amino acid composition of the protein was found to be 81.83 on average, which is conducive to the thermal stability of the spherical protein. The instability index ranged from 35.08 (*ClGRAS31*) to 62.62 (*ClGRAS25*), most of which were associated with unstable proteins; it is noteworthy that the instability indices of *ClGRAS5*, *ClGRAS20*, *ClGRAS31*, *ClGRAS37* and *ClGRAS39* were less than 40, which would indicate that these five proteins were stable. The average hydrophilicity (GRAVY)value of all 43 genes was less than 0, indicating that they are all hydrophilic proteins. Finally, we predicted the subcellular localization of the 43 *ClGRAS* genes using the Plant-mPLoc online tool (version 2.0), which showed that all of them were localized in the nucleus (Table 1).

### 2.2. Phylogenetic Analysis of Chinese Fir GRAS Gene Family

**Figure 1:**
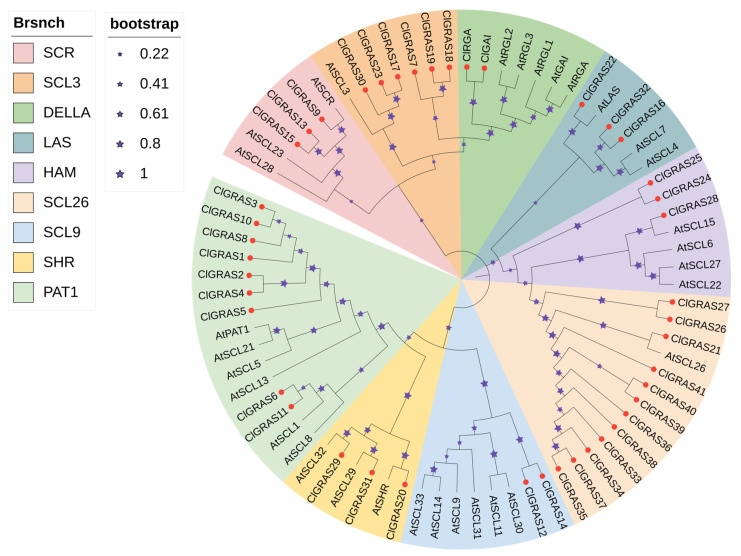
Phylogenetic evolutionary tree of *A. thaliana* and Chinese fir GRAS gene families. Nine colors represent different subfamilies, red dots indicate *ClGRAS* genes, and purple stars represent bootstrap.

In order to gain insight into the evolutionary relationships and classification of the members of the Chinese fir *GRAS* gene family, we constructed a phylogenetic evolutionary tree containing 43 *ClGRAS* members and 32 *A. thaliana AtGRAS* members (Supplementary Material, Table S1) using the NJ method in MEGA software. As shown, we classified the *GRAS* genes into nine subfamilies based on the subfamily classification of GRAS gene families in *A. thaliana*, namely SCR, SCL3, DELLA, LAS, HAM, SCL26, SCL9, SHR, and PAT1 (Figure 1). Of these, the SCL26 subfamily was the most numerous, with 12 members. This was followed by the PAT1 and SCL3 subfamilies with nine and six members, respectively. The DELLA and SCL9 subfamilies were the smallest, comprising only two *ClGRAS* genes each.

### 2.3. Analysis of Gene Structure and Conserved Motifs in ClGRAS Family of Chinese Fir

**Figure 2:**
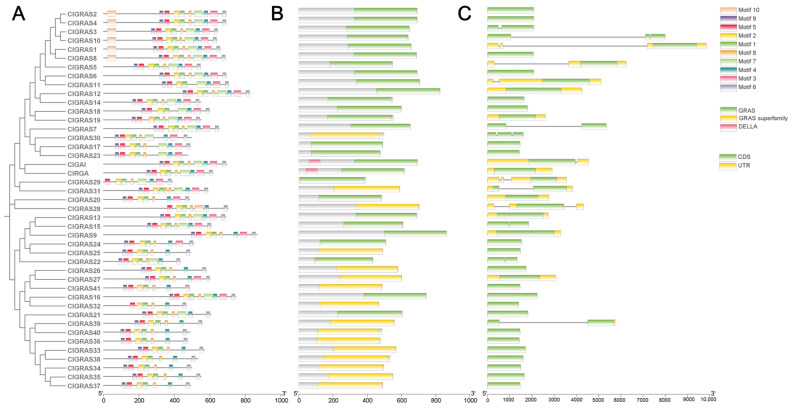
Motif and gene structure analysis of the *ClGRAS* gene family. (**A**) Conserved motifs of *ClGRAS* genes. The phylogenetic evolutionary tree of the 43 *ClGRAS* genes is shown on the left. Different colored boxes on the right represent different motifs and their positions in the GRAS protein sequences. (**B**) Structural domain analysis of ClGRAS proteins. Green is the GRAS family structural domain; yellow is the GRAS superfamily structural domain; pink is the DELLA family structural domain. (**C**) The gene structure of *ClGRAS* genes. Exons are represented as green rectangles. Yellow color represents introns.

To gain further insight into the sequence characterization of ClGRAS proteins, we conducted an in-depth analysis of the motif composition of these proteins via the MEME website. The results demonstrated that among the ten identified motifs, the conserved motifs 1, 2, 4, 5, 6, 8, and 9 exhibited high conservation and were present in 95% of the protein sequences (Figure 2A). It is notable that the number of motifs present in the C-terminal region exceeds those observed in the N-terminal region. The characteristics of the 10 motifs are listed in Figure S1. Moreover, even members of the same subfamily exhibit some variation in the range and frequency of motif distribution. For instance, within the PAT1 subfamily, six genes contain motif 10, while the remaining three (*ClGRAS5, ClGRAS6*, and *ClGRAS11*) lack this motif. Additionally, motif 3 is observed only once in *ClGRAS11*, but twice in each of the other seven genes (Figure 1 and Figure 2A). This indicates that the *ClGRAS11* gene may be implicated in particular functions within the PAT1 subfamily. Subsequently, the conserved structural domains of 43 protein sequences were analyzed using the Batch-CDD tool (version 3.21) from the NCBI website. As illustrated in Figure 2B, all ClGRAS proteins possess GRAS or GRAS superfamily structural domains. It is noteworthy that the DELLA subfamily is the sole one to possess a distinctive DELLA structural domain (Figure 2B). The different structural domains all play crucial roles in the signaling process [30]. Given the pivotal role of gene structural diversity in the evolution of gene families, we undertook an analysis of the intron–exon distribution of *ClGRAS* genes. It was observed that 28 *ClGRAS* genes exhibited no introns, while the number of introns in the remaining 15 genes ranged from two to four (Figure 2C).

### 2.4. Analysis of Cis-Acting Elements and Chromosomal Localization in the ClGRAS Family of Chinese Fir

**Figure 3:**
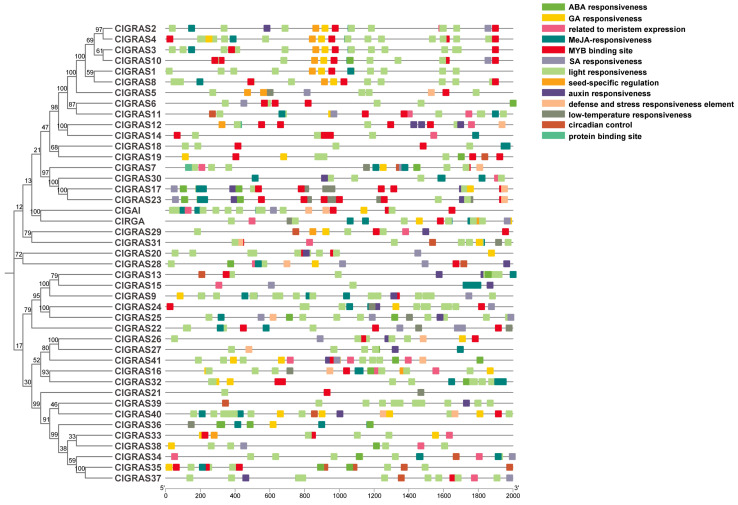
The distribution of cis-acting elements in the 2000 bp promoter upstream of the *ClGRAS* genes. The phylogenetic evolutionary tree of *ClGRASs* is shown on the left, and different colored rectangles in the figure represent different cis-acting elements.

In order to identify potential cis-acting elements and to further investigate the regulatory functions of *GRAS* genes, the 2000-base pair promoter region upstream of 43 *ClGRAS* genes was analyzed using the PlantCARE online tool (https://bioinformatics.psb.ugent.be/webtools/plantcare/html/, access on 15 May 2024). As illustrated in Figure 3, light-responsive elements were identified in the majority of genes. Hormone-associated cis-acting elements include abscisic acid (ABA), GA, Methyl jasmonate (MeJA), SA, and indole acetic acid (IAA). It is noteworthy that, with the exception of the DELLA subfamily, where GA-responsive elements are present in the promoters of both genes, *ClGAI* and *ClRGA*, the majority of genes are only present in the other eight subfamilies. To illustrate, in the PATI family, six out of the eight genes were found to contain this element, while it was absent from the promoters of *ClGRAS5* and *ClGRAS6* (Figure 1 and Figure 3). Nevertheless, the IAA response element is present only in *ClGRAS2* in this subfamily, with the other genes lacking this response element. In contrast, the promoters of the majority of *ClGRAS* genes exhibited the presence of ABA- and MeJA-responsive elements, while SA-responsive elements were identified in only a limited number of promoters. Furthermore, some environment-responsive elements, such as low-temperature-responsive elements, were identified. This type of element is predominantly observed in the SCL3 and HAM families, which may be attributed to the capacity of the genes within this subfamily to engage in temperature-related biological processes. Additionally, meristematic organization-related elements are present, particularly in the promoters of SCL9 and SCL26 subfamily genes. In light of these findings, it can be posited that *ClGRAS* genes may serve a pivotal function in the growth and development of Chinese fir, as well as in its stress response.

**Figure 4:**
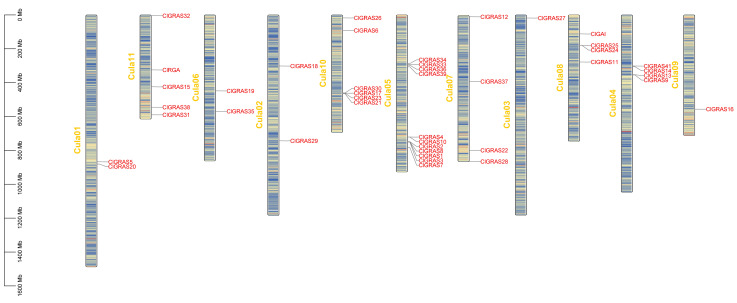
Illustrates the chromosomal distribution of members of the *ClGRAS* gene family. The vertical bars indicate chromosomes, with the chromosome number in yellow on the left side of each bar, the gene name in red on the right side, and the chromosome density in the center. The scale on the left indicates chromosome length.

The genes on the chromosomes were visualized using TBtools software (version 2.119) based on the annotation files of the Chinese fir genome, which facilitated our understanding of the distribution of *ClGRAS* genes on the chromosomes and the genome-wide density (Figure 4). The results demonstrated that 42 genes were not uniformly distributed across the 11 chromosomes of the Chinese fir genome. Additionally, *ClGRAS40* was not localized to any specific chromosome. Of the *ClGRAS* genes, chromosome 05 exhibited the greatest distribution, with 11 genes identified. Subsequently, chromosomes 10 and 11 exhibited a distribution of six and five genes, respectively. The remaining eight chromosomes demonstrated a range of one to four genes. In particular, gene clusters are formed on chromosomes 1, 4, 5, 8, and 10, and functional similarities may exist between genes belonging to the same cluster.

### 2.5. Collinearity Analysis in the ClGRAS Family of Chinese Fir

**Figure 5:**
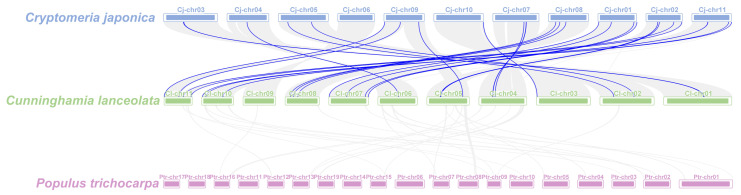
Collinearity analysis of *GRAS* genes between *C. japonica* (blue), Chinese fir (green), and *P*. *trichocarpa* (purple). Gray lines indicate collinear blocks in genomes of Chinese fir and other plants, and blue lines indicate syntenic *GRAS* gene pairs.

The analysis of collinearity among species can provide further insight into phylogenetic mechanisms. Consequently, we conducted an analysis of the collinearity of *GRAS* genes in Chinese fir, *Cryptomeria japonica*, and *Populus trichocarpa*. The results showed that among the woody plants, Chinese fir had a higher rate of *GRAS* gene pairing with Japanese cedar, which is also a Cupressaceae, with a total of 29 pairs of genes (Figure 5). Conversely, no pairing was observed between the *P*. *trichocarpa GRAS* genes and the Chinese fir. This indicates that the Chinese fir and *C. japonica* are more closely related in evolutionary terms.

### 2.6. Sequence Comparison, Conserved Motifs, and Structural Domains Analysis of Chinese Fir DELLA Proteins

**Figure 6:**
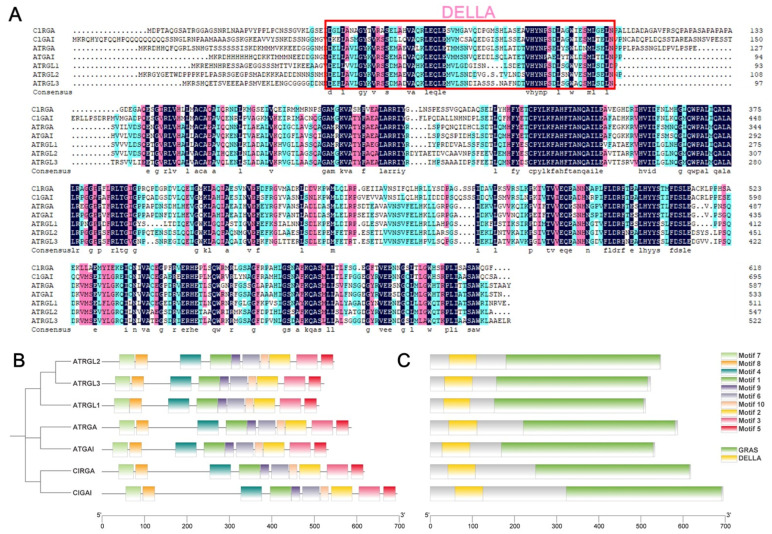
(**A**) A sequence comparison of *ClDELLAs* and *AtDELLAs*. The pink box indicates the DELLA structural domain. (**B**) The distribution of conserved motifs of *ClDELLAs* and *AtDELLAs*. The phylogenetic evolutionary tree of the two is shown on the left. A total of 10 conserved motifs were identified, which are indicated by different colors. (**C**) Conserved structural domains of *ClDELLAs* and *AtDELLAs*. A yellow color indicates DELLA family structural domains and a green color indicates GRAS family structural domains.

The Chinese fir ClGRAS gene family comprises nine subfamilies, exhibiting functional variability among each subfamily. In the present study, we elected to explore the functions of two genes within the DELLA subfamily: *ClGAI* and *ClRGA*. A comparative analysis of the protein sequences of two Chinese fir DELLA proteins and five *A. thaliana* DELLA proteins was conducted. As illustrated in Figure 6A, there was a notable degree of similarity between the two species. To gain further insight into the DELLA protein sequences, we also undertook a detailed characterization of their conserved motifs and structural domains. A total of 10 conserved motifs were identified (Figure 6B), and the results demonstrated a high degree of similarity between the conserved motifs of the ClDELLA protein and those of the *A. thaliana* protein sequences. Both exhibited the presence of the GRAS and DELLA structural domains (Figure 6A,C), indicating that the functions of the DELLA proteins of the two may exhibit some degree of similarity.

### 2.7. Subcellular Localization and Transcriptional Activation Activity of ClDELLA Protein

**Figure 7:**
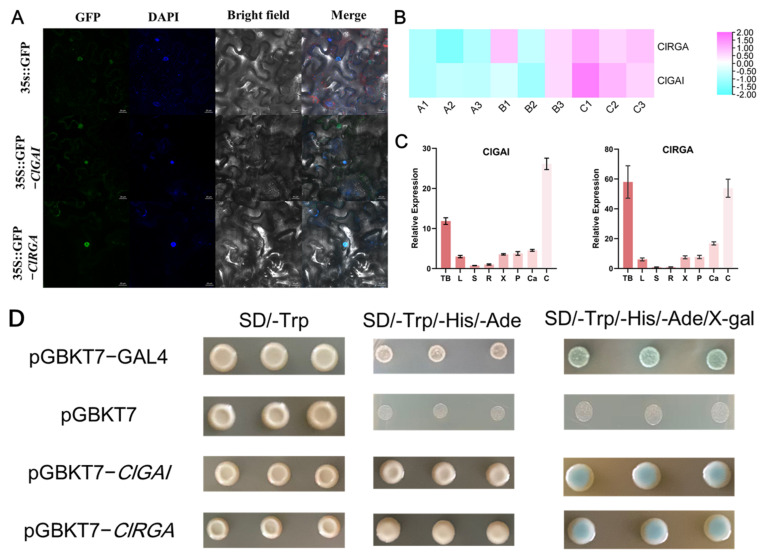
Expression patterns and transient expression analysis of *ClGAI* and *ClRGA*. (**A**). Subcellular localization of ClDELLAs in tobacco leaves. The expression of *ClGAI* and *ClRGA*, which have been fused with GFP tags, was observed in tobacco leaves. The image is composed of four columns, each representing a different set of data: GFP, DAPI, bright field, and these images have been merged to create a single, composite representation. Bar = 25 μm. (**B**) Transcriptional profiles of Chinese fir *DELLA* genes under different developmental stages. A1-A3, B1-B3, and C1-C3 represent the fir growth reactivation, active, and dormant phases, respectively. The generation of heatmaps was based on log2 (FPKM + 1) values, which were subsequently normalized to a line scale. The color scales indicate the relative expression levels. (**C**) The relative expression levels of *ClDELLAs* in different tissues of the fir are presented. tb, terminal bud; L, leaf; S, stem; R, root; X, xylem; P, phloem; Ca, cambium; C, cones. (**D**) The positive control, negative control, pGBKT7-*ClGAI,* and pGBKT7-*ClRGA* yeast cells were incubated in SD/-Trp, SD/-Trp-His-Ade, and SD/-Trp-His-Ade + X-α-gal medium, respectively.

We explored the fundamental properties of transcription factors through nuclear localization and transcriptional activation. The results of subcellular localization are shown in Figure 7A, where *ClGAI* and *ClRGA* are localized in the nucleus, consistent with the predicted results from bioinformatics analysis of *ClGAI* and *ClRGA* proteins (Table 1). To investigate the transcriptional activation activity of the two genes, the full-length CDS was fused to the DNA-binding domain of GAL4 (GAL4-BD) within the pGBKT7 vector, and the resulting construct was introduced to the yeast strain AH109. The AH109 strain was obtained by screening on tryptophan-free SD medium (SD/-Trp). Transformants containing the pBD-*ClDELLA* construct exhibited a blue coloration on SD medium containing X-α-gal but not Trp, His, and Ade (SD/-Trp-His-Ade), whereas the negative control displayed no coloration (Figure 7D). This suggests that both *ClGAI* and *ClRGA* possess transcriptional activation activity.

### 2.8. Expression Pattern of ClGAI and ClRGA in Different Tissues

It has been shown that in *A. thaliana* and populus, DELLA proteins can directly or indirectly affect the stem vascular cambium [31]. Therefore, to further understand the function of *ClDELLA* genes, we first analyzed two *DELLA* genes by real-time quantitative PCR (qRT-PCR). The results demonstrated that both genes were expressed in a majority of organs and tissues, including terminal buds, leaves, stems, roots, phloem, xylem, the vascular cambium, and cones. However, the expression levels exhibited considerable variation (Figure 7C). Both genes were highly expressed in terminal buds and cones, followed by the vascular cambium, while lower expression was found in stems and roots. This suggests that the expression of *ClGAI* and *ClRGA* is spatiotemporally specific during the growth and development of Chinese fir. However, the results of the transcriptomic analysis of the vascular cambium of Chinese fir at different developmental stages (reactivation (April), active (July), and dormant (September)) demonstrated that the expression levels of the two genes exhibited a gradual increase over time (Figure 6B). This finding is in accordance with the results presented in Figure 7C.

### 2.9. Expression Pattern Analysis of ClDELLAs Under Different Hormonal and Abiotic Stresses

**Figure 8:**
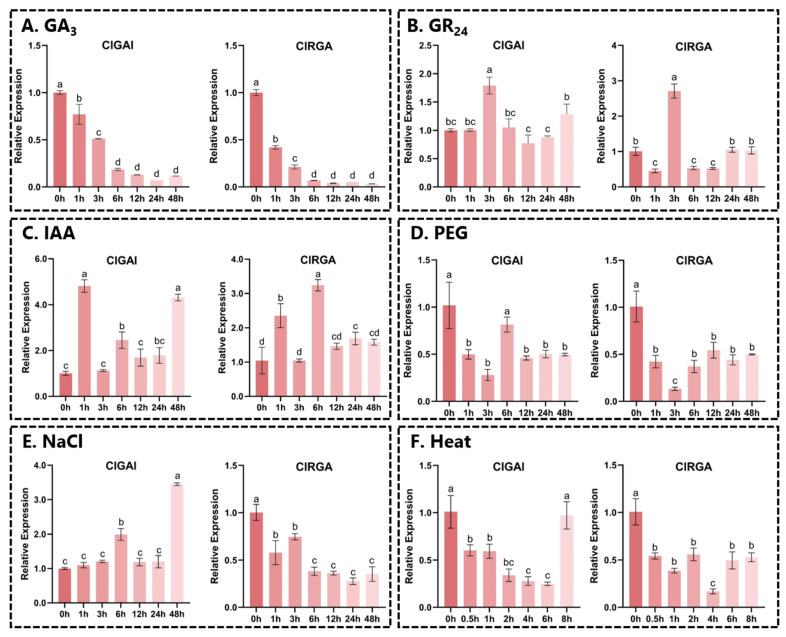
Relative expression of *ClDELLA* genes under different treatments. (**A**) GA_3_, (**B**) GR_24_, (**C**) IAA, (**D**) PEG, (**E**) NaCl, and (**F**) heat. Vertical coordinates are relative expressions and horizontal coordinates indicate different sampling times. Same lowercase letters between different columns indicate that differences are not significant. Highest column is marked with “a”, then “b”, and so on. Completely different lowercase letter between columns indicates significant difference; *p* < 0.0001.

To gain further insight into the functions of *ClGAI* and *ClRGA*, we conducted RT-qPCR analysis to examine the expression patterns of *ClDELLAs* in two-year-old Chinese fir seedlings subjected to various hormonal and abiotic stresses (Figure 8). It is noteworthy that under 200 μM GA_3_ treatment, the expression of both genes showed a gradual decrease with increasing time. This demonstrates the response of the *DELLA* genes to GA stress. Under 20 μM strigolactone (GR_24_) treatment, the expression of both *ClGAI* and *ClRGA* significantly increased at 6 h, and then gradually increased at 24 h. This phenomenon may be attributed to a gradual decline in GR_24_ hormone levels over time. In addition, both genes could be induced by IAA at different time points. Under 15% (*w*/*v*) PEG stress treatment, the expression of both genes was significantly decreased at 6 h, and both were repressed. In contrast, under 200 mM NaCl stress, the two genes showed different expression trends, with *ClGAI* expression increasing while *ClRGA* expression was suppressed. After the application of heat stress, the expression of both genes was found to be significantly decreased, but the expression of *ClGAI* increased at 8 h, and we speculate that it might be induced by other genes.

## 3. Discussion

The GRAS family plays a pivotal role in the signaling pathways of plants [32], as well as in their growth and development. However, the characterization and expression patterns of GRAS genes involved in abiotic stress responses in Chinese fir have not been previously elucidated. Consequently, we identified and analyzed *GRAS* genes in Chinese fir, and two *DELLA* genes were selected for functional analysis under different abiotic stress conditions.

In the present study, a total of 43 *GRAS* genes were identified in Chinese fir based on a whole genome thereof, and they were classified into nine subfamilies based on the *A. thaliana* subfamily classification. These subfamilies were as follows: SCR, SCL3, DELLA, LAS, HAM, SCL26, SCL9, SHR, and PAT1 (Figure 1). The number of GRAS family members in Chinese fir (43), similar to that of *Betula platyphylla* (40) [33], is higher than that of *A. thaliana* (33) [8], but less than that of *Cyclocarya paliurus* (51) [34], *Castanea mollissima* (48) [35], and *Malus domestica* (127) [36]. Variations in the number of gene families may be linked to genome size or gene duplication events during evolutionary processes [34]. Furthermore, the combination of conserved motifs, structural domains, and gene structures demonstrated similarities among genes within the same subfamily and differences among those in different subfamilies. It is noteworthy that the PAT1 family was the only one to contain motif 10 (Figure 2A), a result that aligns with the motifs of *Glycine max* [37] and *Symplocos tanakana* [38], but differs from those of *cassava* [13], *E. grandis* [12], and *Cymbidium goeringii* [39]. For instance, an examination of the GRAS family of *E. grandis* [12] reveals that the PAT1 subfamily exhibits a comparable motif composition and distribution. However, within the Chinese fir GRAS family, PAT1 is observed to occur with a motif 10 that is not present in other families. The presence of specific motifs in proteins indicates that they have unique functions. This indicates that the genes within the Chinese fir PATI subfamily may possess distinctive functions. It is noteworthy that the DELLA structural domain is exclusive to the DELLA family, while other subfamilies encompass the GRAS family or the GRAS superfamily. This may contribute to the diversification of the GRAS gene family and influence its functional specialization. An analysis of exon–intron patterns can provide further insights into the evolution of gene families. The gene structure of the ClGRAS family revealed that 28 genes lacked introns, while the remaining 15 genes had introns ranging from two to four (Figure 2C). The presence of introns serves to mitigate the deleterious effects of mutations on coding sequences. It has been demonstrated that the primary cause of intron deletion in gene families is rapid replication at the bacterial level following horizontal gene transfer [40].

Cis-acting elements are transcription factor binding sites and other regulatory motifs present in the paralogous sequences of genes with specific functions [41]. These elements are of significant importance with regard to the processes of gene transcription and regulation. In this study, we have identified cis-acting elements within the 2000 bp promoter region upstream of the *ClGRAS* genes. (Figure 3). The results of our investigation demonstrated the pervasive occurrence of phytohormone-responsive elements (SA, GA, MeJA, etc.) and abiotic stress elements (defense, low temperature, drought, etc.). Additionally, we observed the occurrence of elements related to plant growth and development, including meristematic tissue expression. These components were also commonly found with GRAS family genes in woody plants such as *B. platyphylla* [33] and *Larix kaempferi* [42], as well as in herbaceous plants such as *C*. *macrorrhizum* [39] and soybean [37]. This suggests that there is a similarity in the function of the GRAS gene family across species. It is noteworthy that the Chinese fir GRAS family contains some differences in the elements among the different subfamilies. To illustrate, the DELLA subfamily functions as a gibberellin response factor and comprises genes that all contain GA elements; the low-temperature response elements are predominantly found in the SCL3 and HAM families, indicating that they may be involved in the regulation of responses to temperature changes; and the majority of meristem-organization-related elements are concentrated in the SCL9 and SCL26 subfamilies, which suggests that they may have a regulatory function with regard to growth. To gain further insight into the characteristics of the ClGRAS family of genes, we conducted a chromosomal localization analysis on 43 genes. The results indicated that 42 of the genes were unevenly distributed across 11 chromosomes, while *ClGRAS40* was not localized on any chromosomes.

Due to its rapid growth and superior wood properties, Chinese fir has become the most widely cultivated gymnosperm [43] in southern China. Consequently, a comprehensive understanding of its growth and development mechanisms is of paramount importance. In the GRAS family, the DELLA protein inhibits the mitotic activity of poplar cambium cells by interacting with *ARK2* and *WOX4*, key regulators of the vascular cambium, and affecting the binding of *ARK2* and *WOX4* to downstream target genes [27]. Additionally, the DELLA protein is responsible for the formation of the SCR-SHR-DELLA complex, which plays a pivotal role in the circumferential morphogenesis of roots and stems [44]. Accordingly, an investigation of the *DELLA* gene in Chinese fir was deemed appropriate. The initial step involved an examination of the expression levels of *ClGAI* and *ClRGA* in various tissues. The findings indicated that these genes were implicated in the diverse stages of growth and development of Chinese fir. The elevated expression of the two genes in the vascular cambium indicates the potential involvement of these genes in the secondary growth of fir. Furthermore, the transcript levels of *DELLA* genes were analyzed in the vascular cambium at different developmental stages. The expression levels of *DELLA* genes demonstrated a gradual increase over time, with the highest transcript levels observed during the dormant stage. This indicates the potential involvement of *DELLA* genes in the response to adversity. However, further analyses and experiments are necessary to substantiate this hypothesis.

It has been demonstrated that DELLA proteins play a significant role in plant signaling pathways and stress response [20]. The expression of *DELLA* genes varies in response to abiotic stress in plants [45]. To illustrate this, salt stress or low-temperature stress has been observed to regulate the transcript abundance of specific *DELLA* genes in *Cucurbita moschata* [46]. Similarly, our findings indicated that both genes were repressed in response to GA treatment, drought stress, and high-temperature stress. Conversely, they were induced in IAA treatment. However, the two genes exhibited disparate trends in GR and NaCl treatments [47]. The aforementioned results indicate that *ClGAI* and *ClRGA* are integral to the response to abiotic stresses. For the proteins to perform their functions, they must be localized in appropriate regions. The results of subcellular localization showed that *ClGAI* and *ClRGA* play a regulatory role in the nucleus, which is consistent with the predicted results and with the localization of *DELLA* genes in *A. thaliana* [8], *Vaccinium darrowii* [48], poplar [14], and *Paulownia fortune* [49]. Moreover, both genes demonstrated transcriptional activation activity. Further investigation of their downstream regulatory genes may provide insights into the molecular mechanisms of growth and development and stress resistance in Chinese fir. As GA-negative regulators [50], they exhibited repression under GA treatment and demonstrated disparate responses to GR and IAA. Both genes exhibited repression under conditions of drought and high-temperature stress; however, they demonstrated disparate trends under salt treatment, indicating that they may serve as promising candidates for further investigation into their abiotic resistance. It is noteworthy that this study represents the inaugural report of *GRAS* genes in Chinese fir, has augmented the genetic data pertaining to the ClGRAS gene family, and has established a foundation for future investigations into the functional roles of *GRAS* genes, particularly the DELLA protein. Furthermore, this study offers a theoretical foundation for the selection and improvement of forest trees.

## 4. Materials and Methods

### 4.1. Identification and Physicochemical Characterization of Chinese Fir GRAS Gene Family

The Hidden Markov Model (HMM) profile for the GRAS domain (PF03514) was obtained from the Pfam database (Pfam is now hosted by InterPro (xfam.org)). The Chinese fir genome files were sourced from the NCBI website (National Center for Biotechnology Information (nih.gov)). The GRAS protein sequences from *Arabidopsis* were obtained from the TAIR website (TAIR–Home (arabidopsis.org)). The Hidden Markov Model (HMM) was employed as a query in the TBtools software to identify potential *ClGRAS* genes within the Chinese fir genome. A total of 43 candidate *ClGRAS* genes were identified and their protein sequences were extracted. The physicochemical properties of the candidate proteins were subsequently analyzed and plotted in a table using the online tool ExPasy (SIB Swiss Institute of Bioinformatics|Expasy) (Table 1). Furthermore, the subcellular localization of the candidate genes was predicted using the Plant-mPLoc (Plant-mPLoc server (sjtu.edu.cn) online tool).

### 4.2. Phylogenetic Analysis of the Chinese Fir GRAS Gene Family

Phylogenetic trees were constructed using *Arabidopsis* and Chinese fir GRAS protein sequences in MEGA 7.0 software, employing the neighbor-joining (NJ) method and 1000 bootstrap test replicates.

### 4.3. Analysis of Gene Structure and Conserved Motifs in the ClGRAS Family of Chinese Fir

The gene structure was analyzed using TBtools software, based on the Chinese fir genome annotation file (GFF3). The structural domains of 43 *ClGRAS* genes were then predicted by the Batch-CDD (Welcome to NCBI Batch CD-search (nih.gov)) online tool of the NCBI database, utilizing the default parameters. The conserved motifs of the GRAS proteins were analyzed using the MEME (Introduction–MEME Suite (meme-suite.org)) online tool (version 5.5.7), which revealed significant differences between members of the ClGRAS family. In conclusion, the aforementioned results were visualized using TBtools software (version 2.119).

### 4.4. Analysis of Cis-Acting Elements and Chromosomal Localization in the ClGRAS Family of Chinese Fir

In total, 2000 base pair sequences located upstream of each gene were extracted from the Chinese fir genome annotation file (GFF3) using TBtools software. These sequences were subsequently analyzed for promoter cis-acting elements using the online analysis software PlantCARE (PlantCARE, a database of plant promoters and their cis-acting regulatory elements (ugent.be)). The results were then filtered and simplified, and a visual representation was generated using TBtools software. Chromosome position data were received from the genome annotation file (GFF3) and mapped using TBtools software.

### 4.5. Expression Pattern Analysis of ClGAI and ClRGA

Thirty-year-old fir trees exhibiting robust growth were selected as sample materials for vascular cambium RNA-seq. The vascular cambium bands were obtained by scraping the stems. Samples were collected at three distinct growth phases: reactivation, active, and dormant. These samples were designated as A, B, and C, respectively. The samples were promptly placed in a freezer set to −80 °C for the purpose of RNA isolation. In addition, the two-year-old Chinese fir seedlings utilized in this study were sourced from Yangkou Forestry in Fujian, China, and subsequently transplanted to the greenhouse of Nanjing Forestry University for a two-month period of acclimatization. We collected RNA from organs such as terminal buds, leaves, stems, and roots of Chinese fir in April and tissues such as phloem, xylem, and vascular cambium as well as cones during the period of vigorous growth (July) (AG21019, ACCURATE BIOTECHNOLOGY (HUNAN), Co., Ltd., Changsha, China).

Healthy Chinese fir seedlings of equivalent height were selected and subjected to distinct treatments in a separate manner. For the purposes of this study, seedlings were selected for individual treatment with a solution of 200 μM GA3, 50 mg/L IAA, and 20 μM GR24 until the needle surfaces were completely wetted [51]. To simulate drought stress and salt stress, 15% (*w*/*v*) PEG-6000 and 200 mM NaCl were used as osmotic stressors, while a continuous 40 °C treatment was employed to imitate high-temperature stress [52]. Samples were collected at 0, 1, 3, 6, 12, 24, and 48 h, with the exception of those subjected to high-temperature stress, which were immediately stored at −80 °C for RNA isolation. Sampling was conducted at 0, 0.5, 1, 2, 4, and 8 h for the high-temperature stress treatment [53]. Three biological and three technical replicates were conducted for each treatment. Following the extraction of RNA from the samples, cDNA was generated through reverse transcription (SPARKscriptⅡRT Plus Kit (With gDNA Eraser) (Shandong Sparkjade Biotechnology Co., Ltd., Jinan, China)). Real-time quantitative PCR primers (Supplementary Material, Table S2) were then designed and tested using the Primer 6.0 software, The reaction was performed through the 2x Realab Green PCR Fast mixture (Beijing LABLEAD Inc., Beijing, China) in a 96-Well PCR plate (2040101, SAINING Biotechnology, Suzhou, China) with the quality of the PCR reactions evaluated from the melting curves. Three independent biological replicates and three technical replicates were tested for each biological replicate. A comparative cycle threshold (Ct) was employed for the quantification of gene expression levels, which were calculated as 2 (−∆Ct) [∆Ct = Ct Target − Ct TUA]. The results were then visualized and marked for significance using GraphPad software (version 10.1.2).

### 4.6. Subcellular Localization of the ClDELLA Protein

The cDNA sequence of the *ClDELLA* gene was extracted from the Chinese fir genome file using TBtools software. The coding sequences (CDSs) of the candidate genes, designated *ClGAI* and *ClRGA*, were cloned using the rapid amplification of cDNA ends (RACE) technique (Cat#10154; Yeasen, Shanghai, China). The enzymatic cleavage sites were selected to be BamHI and EcoRI, and the complete coding sequence was integrated into a modified vector, PCAMBIA1302, which contains a green fluorescent protein (GFP) reporter gene, via homologous recombination (CloneUFO® II One Step Cloning Kit (ATG Biotechnology, Nanjing, China)). Subsequently, the construct was transformed into strain GV3101, which was then used to infest tobacco epidermal cells. The gene localization was determined by observing the fluorescence signal.

### 4.7. Transcriptional Activation Activity of the ClDELLA Protein

The enzyme cleavage sites were selected as BamHI and EcoRI, the intact CDS was inserted into the pGBKT7 vector by homologous recombination, and the construct was introduced into Dh5α (#CC96102, Tolo Biotech Co., Ltd., Shanghai, China) and yeast strain AH109. Screening on tryptophan-free SD medium (SD/-Trp) yielded the AH109-positive strain (28 °C, 48–96 h). Subsequently, yeast cells were cultured on SD/-Trp-His-Ade and SD/-Trp-His-Ade+X-α-gal (28 °C, 48–96 h). The transcriptional activation activity of the proteins was determined by observing the growth of the strains on the medium and the resulting discoloration.

## 5. Conclusions

In the present study, a total of 43 *ClGRAS* genes were identified in the Chinese fir genome, and phylogenetic analyses revealed the existence of nine distinct clades. A gene structure analysis demonstrated that the majority of *ClGRAS* genes lacked introns, indicating that they are highly structurally conserved. The members of the same clade exhibit extensive similarity in conserved motifs and structural domains, which suggests that they have parallel gene functions. Furthermore, an investigation was conducted into the functions of the DELLA subfamily genes. The two genes demonstrated differential expression in discrete tissues, suggesting that they are spatio-temporally specific with regard to their involvement in plant growth and development. Based on the transcriptome data of the vascular cambium of Chinese fir at different developmental stages and RT-qPCR analysis after different treatments, it was found that they could be candidate genes for further study of their abiotic resistance. Importantly, this study reported the *GRAS* genes in Chinese fir for the first time, enriched the genetic information of the ClGRAS gene family, and established a foundation for the investigation of the function of *GRAS* genes, particularly the DELLA protein. Furthermore, it offers a theoretical foundation for the selection and improvement of forest trees.

## Figures and Tables

**Table 1:** Detailed information and physicochemical properties of ClGRASs.

Gene Name	Chromosome Localization	Prediction of Subcellular Localization	Length (aa)	Molecular Weight (kDa)	pI	Aliphatic Index	Instability Index	GRAVY
*ClGRAS1*	Cula05	Nucleus	1316	146.94	5.19	74.98	55.63	−0.432
*ClGRAS2*	Cula05	Nucleus	692	77.33	4.98	71.69	56.07	−0.510
*ClGRAS3*	Cula05	Nucleus	646	72.55	5.15	74.85	52.66	−0.443
*ClGRAS4*	Cula05	Nucleus	692	77.36	4.98	71.69	55.92	−0.495
*ClGRAS5*	Cula01	Nucleus	548	61.25	4.87	83.67	39.28	−0.328
*ClGRAS6*	Cula10	Nucleus	693	77.13	5.02	83.16	54.46	−0.336
*ClGRAS7*	Cula05	Nucleus	652	71.70	5.84	79.19	56.74	−0.327
*ClGAI*	Cula08	Nucleus	695	77.30	5.29	78.62	60.04	−0.480
*ClRGA*	Cula11	Nucleus	618	66.72	5.55	85.66	50.30	−0.103
*ClGRAS8*	Cula05	Nucleus	688	76.81	4.89	74.69	56.44	−0.463
*ClGRAS9*	Cula04	Nucleus	866	94.44	5.79	76.09	48.58	−0.476
*ClGRAS10*	Cula05	Nucleus	639	72.06	5.57	74.90	53.17	−0.470
*ClGRAS11*	Cula08	Nucleus	707	78.21	6.13	86.35	48.55	−0.304
*ClGRAS12*	Cula07	Nucleus	826	92.98	5.76	75.04	49.17	−0.535
*ClGRAS13*	Cula04	Nucleus	691	75.96	5.36	74.43	48.26	−0.364
*ClGRAS14*	Cula04	Nucleus	549	62.21	6.06	84.70	45.36	−0.335
*ClGRAS15*	Cula11	Nucleus	609	67.17	5.69	76.77	56.08	−0.263
*ClGRAS16*	Cula09	Nucleus	746	82.27	5.45	72.12	55.39	−0.353
*ClGRAS17*	Cula10	Nucleus	491	54.87	6.33	86.05	60.54	−0.245
*ClGRAS18*	Cula02	Nucleus	599	67.13	6.45	80.18	59.07	−0.393
*ClGRAS19*	Cula06	Nucleus	552	61.35	5.31	82.07	45.73	−0.298
*ClGRAS20*	Cula01	Nucleus	485	55.56	5.58	70.43	39.75	−0.353
*ClGRAS21*	Cula10	Nucleus	605	67.13	5.69	79.85	49.92	−0.266
*ClGRAS22*	Cula07	Nucleus	433	48.88	5.41	78.48	56.54	−0.470
*ClGRAS23*	Cula10	Nucleus	478	53.39	6.33	86.34	60.06	−0.267
*ClGRAS24*	Cula08	Nucleus	509	57.83	8.39	88.53	48.61	−0.302
*ClGRAS25*	Cula08	Nucleus	492	55.79	8.88	89.21	62.62	−0.375
*ClGRAS26*	Cula10	Nucleus	580	65.18	5.29	87.95	62.29	−0.206
*ClGRAS27*	Cula03	Nucleus	601	67.67	5.61	90.55	62.04	−0.169
*ClGRAS28*	Cula07	Nucleus	704	79.47	5.22	81.99	54.76	−0.314
*ClGRAS29*	Cula02	Nucleus	390	43.72	5.77	85.05	54.84	−0.196
*ClGRAS30*	Cula10	Nucleus	498	56.01	5.68	86.08	61.90	−0.251
*ClGRAS31*	Cula11	Nucleus	590	65.03	5.42	77.10	35.08	−0.455
*ClGRAS32*	Cula11	Nucleus	468	52.05	4.91	83.55	55.18	−0.163
*ClGRAS33*	Cula05	Nucleus	570	64.49	4.62	84.68	46.32	−0.312
*ClGRAS34*	Cula05	Nucleus	497	55.60	4.76	86.36	45.98	−0.239
*ClGRAS35*	Cula06	Nucleus	551	62.12	4.46	86.93	47.96	−0.244
*ClGRAS36*	Cula05	Nucleus	476	53.72	4.53	86.85	46.55	−0.157
*ClGRAS37*	Cula07	Nucleus	491	54.95	4.70	90.81	38.73	−0.192
*ClGRAS38*	Cula11	Nucleus	534	59.21	4.75	89.87	48.72	−0.213
*ClGRAS39*	Cula05	Nucleus	559	62.57	4.61	84.96	36.75	−0.177
*ClGRAS40*	contig09198	Nucleus	488	55.25	4.73	93.93	57.21	−0.039
*ClGRAS41*	Cula04	Nucleus	487	55.12	5.16	82.20	50.01	−0.179

## Data Availability

Data are contained within the article and Supplementary Materials.

## Supplementary Materials

The following supporting information can be downloaded at: https://www.mdpi.com/article/10.3390/ijms252212262/s1.
