# Supplementary material for: Genome-Wide Identification of GRAS Gene Family in Cunninghamia lanceolata and Expression Pattern Analysis of ClDELLA Protein Under Abiotic Stresses
Source: Int J Mol Sci. 2024 Nov 15;25(22):12262. doi: 10.3390/ijms252212262 (PMC11594883; doi:10.3390/ijms252212262)
Supplement: Supplementary file 1 [file ijms-25-12262-s001.zip › ijms-3274456-supplementary.pdf]

Table S1. Gene ID of *A. thaliana* GRAS family

Gene name	Gene ID
AtGAI	At1g14920
AtLAS/SCL18	At1g55580
AtPAT1	At5g48150
AtRGA	At2g01570
AtRGL1	At1g66350
AtRGL2	At3g03450
AtRGL3	At5g17490
AtSCL1	At1g21450
AtSCL3	At1g50420
AtSCL4	At5g66770
AtSCL5	At1g50600
AtSCL6	At4g00150
AtSCL7	At3g50650
AtSCL8	At5g52510
AtSCL9	At2g37650
AtSCL11	At5g59450
AtSCL13	At4g17230
AtSCL14	At1g07530
AtSCL15	At4g36710
AtSCL16	AB007647
AtSCL21	At2g04890
AtSCL22	At3g60630
AtSCL23	At5g41920
AtSCL26	At4g08250
AtSCL27	At2g45160
AtSCL28	At1g63100
AtSCL29	At3g13840
AtSCL30	At3g46600
AtSCL31	At1g07520
AtSCL32	At3g49950
AtSCL33	At2g29060
AtSCR	At3g54220
AtSHR	At4g37650

Table S2. Sequences of Primers

Primer name	Primer sequence
GAI-F	ATGAAGCGACAACACTATCAGTTCC
GAI-R	TCAGGCAGAGCATTGCCAA
RGA-F	ATGGATCCCACCGCTCAAG
RGA-F	CTAAAATCCTTGCCACGCGG
YXB-ClGAI-F	tatgaccatgattacgaattcATGAAGCGACAACACTATCAGTTCC
YXB-ClGAI-R	caggtcgactctagaggatccTCAGGCAGAGCATTGCCAA
YXB-ClRGA-F	tatgaccatgattacgaattcATGGATCCCACCGCTCAAG
YXB-ClRGA-R	caggtcgactctagaggatccCTAAAATCCTTGCCACGCGG
BD-ClRGA-F	atggccatggaggccgaattcATGGATCCCACCGCTCAAG
BD-ClRGA-R	ccgctgcaggtcgacggatccCTAAAATCCTTGCCACGCGG
BD-ClGAI-F	atggccatggaggccgaattcATGAAGCGACAACACTATCAGTTCC
BD-ClGAI-R	ccgctgcaggtcgacggatccTCAGGCAGAGCATTGCCAA
ClGAI-F	CCCAGATGTGGAACTTGAGCG
ClGAI-R	CGCCACGGGTAGACGGTTTT
ClRGA-F	GCAAACTTCCACCTCACAG
ClRGA-R	GGACCTTCACAGGCAACTA
Actin-F	GAGGGACCAGATTCATCGTATTC
Actin-R	ATGCTGGTATTGCTGATCGTATG

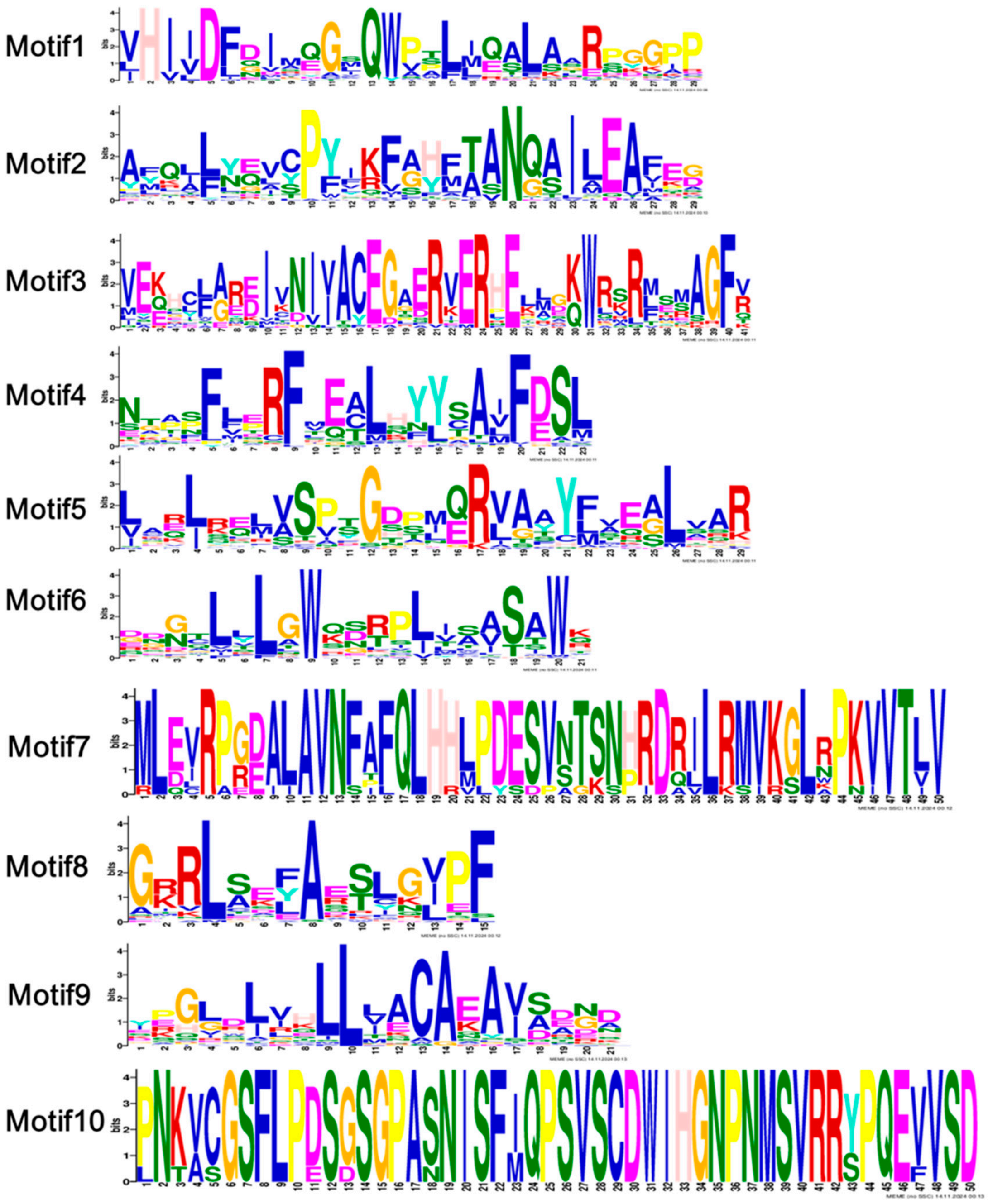

Figure S1. Conserve motif sequence information. The motifs identified as 1-10 are consistent with those illustrated in Figure 2A.
